# Receptor for advanced glycation endproducts (RAGE) maintains pulmonary structure and regulates the response to cigarette smoke

**DOI:** 10.1371/journal.pone.0180092

**Published:** 2017-07-05

**Authors:** Lisa Wolf, Christian Herr, Julia Niederstraßer, Christoph Beisswenger, Robert Bals

**Affiliations:** Department of Internal Medicine V – Pulmonology, Allergology and Critical Care Medicine, Saarland University, Homburg, Germany; University of Rochester Medical Center, UNITED STATES

## Abstract

The receptor for advanced glycation endproducts (RAGE) is highly expressed in the lung but its physiological functions in this organ is still not completely understood. To determine the contribution of RAGE to physiological functions of the lung, we analyzed pulmonary mechanics and structure of wildtype and RAGE deficient (RAGE^-/-^) mice. RAGE deficiency spontaneously resulted in a loss of lung structure shown by an increased mean chord length, increased respiratory system compliance, decreased respiratory system elastance and increased concentrations of serum protein albumin in bronchoalveolar lavage fluids. Pulmonary expression of RAGE was mainly localized on alveolar epithelial cells and alveolar macrophages. Primary murine alveolar epithelial cells isolated from RAGE^-/-^ mice revealed an altered differentiation and defective barrier formation under *in vitro* conditions. Stimulation of interferone-y (IFNy)-activated alveolar macrophages deficient for RAGE with Toll-like receptor (TLR) ligands resulted in significantly decreased release of proinflammatory cytokines and chemokines. Exposure to chronic cigarette smoke did not affect emphysema-like changes in lung parenchyma in RAGE^-/-^ mice. Acute cigarette smoke exposure revealed a modified inflammatory response in RAGE^-/-^ mice that was characterized by an influx of macrophages and a decreased keratinocyte-derived chemokine (KC) release. Our data suggest that RAGE regulates the differentiation of alveolar epithelial cells and impacts on the development and maintenance of pulmonary structure. In cigarette smoke-induced lung pathology, RAGE mediates inflammation that contributes to lung damage.

## Introduction

The receptor for advanced glycation endproducts (RAGE) is a cell surface receptor and belongs to the immunoglobulin superfamily [1]. Under physiological conditions, RAGE is highly expressed in the lung, while in other organs and tissues its expression rate is very low [2]. The pulmonary localization of RAGE is mainly attributed to alveolar epithelial cells [2, 3]. In these cells, RAGE is involved in the cell adhesion to extracellular matrix proteins and supports the characteristic cell-specific morphology by enhancing their spreading and flattening [2].

RAGE is also a pattern-recognition receptor that interacts with a broad range of ligands like the DNA binding protein high mobility group box 1 (HMGB1) [4], S100 proteins [5], advanced glycation endproducts (AGEs) [6] and beta amyloid proteins [7]. Depending on the cell type and ligand, RAGE-ligand-interactions stimulate multiple series of cellular signaling pathways and lead to activation of different transcription factors such as NF-κB, activator protein-1 (AP-1), cAMP response element binding protein (CREB), or signal transducers and activators of transcription 3 (STAT-3) [8–12]. These transcription factors promote the production of proinflammatory mediators, adhesion molecules and importantly RAGE itself [11, 13, 14]. As a consequence, RAGE is up-regulated at sites where its ligands accumulate and causes a sustained inflammatory response resulting in chronic inflammation [11].

Chronic obstructive pulmonary disease (COPD) is a progressive lung disease characterized by chronic airway inflammation leading to alveolar tissue destruction and airflow limitation [15]. Cigarette smoking is a major risk factor for the development of COPD, while also exposure to other sources of smoke can induce COPD [16]. Long-term exposure to cigarette smoke (CS) results in the release of damage-associated molecular patterns (DAMPs) such as HMGB1 or S100 proteins [17]. CS contains reactive glycation products that react nonenzymatically with proteins, lipids and nucleic acids to produce AGEs [18]. In the lung tissue of smokers and patients with COPD, immunohistochemical staining showed increased levels of AGEs [19, 20]. HMGB1 concentrations in bronchoalveolar lavage fluid (BALF) and the amount of HMGB1-positive macrophages and epithelial cells in bronchial biopsies and lung tissue sections were also increased in COPD patients compared to never-smokers [21]. These studies also demonstrated a stronger staining intensity for RAGE in pulmonary tissue [20, 21], which was associated with a decline of FEV_1_ [20]. In an elastase-induced emphysema-model, RAGE knockout mice were partly protected from structural lung changes and this effect was mainly attributed to the absence of RAGE in lung structural cells [22]. Mice exposed to acute secondhand smoke showed an increase in pulmonary RAGE protein expression [23]. Moreover, CS-induced release of proinflammatory cytokines and pulmonary recruitment of inflammatory cells were inhibited in RAGE^-/-^ mice exposed to secondhand smoke [23] or cigarette smoke extract [24].

The purpose of this study was to determine the role of RAGE in normal lung development and maintenance of homeostasis by analyzing cell type specific functions of RAGE. We further investigated the participation of RAGE in CS induced inflammation and in the development of emphysema.

## Material and methods

### Animal studies

All animal experiments were approved by the Landesamt für Soziales, Gesundheit und Verbraucherschutz of the State of Saarland following the national guidelines for animal treatment. Mice were maintained under pathogen-free conditions. RAGE deficient C57BL/6N (RAGE^-/-^) mice were kindly provided by Prof. Arnold and Prof. Nawroth from the German Cancer Research Center (DKFZ) and University Clinical Center of Heidelberg, respectively [25].

### Short-term cigarette smoke exposure

We used two different CS exposure models with different outcomes. The focus of the short-term model is on the development of acute, neutrophilic inflammation, the focus of the long-term model is on structural changes. Eight week old female wildtype (WT) and RAGE^-/-^ mice were analyzed in a short-term smoking system [26]. In short, mice were exposed to smoke from eight unfiltered cigarettes (3R4F, College of Agriculture, Reference Cigarette Program, University of Kentucky, Lexington, Kentucky, USA) in an acryl glass box for 20–30 minutes. Two mechanical ventilators (7025 rodent ventilator, Ugo Basile, Biological Research Instruments, Comerio, Italy) were used to pump cigarette smoke (675 mL/min) and room-air (1800 mL/min) into the exposure chamber. This procedure was repeated five times a day, for four consecutive days. Between each CS exposure, the mice were removed in their cages for a minimum of one hour. The quality of air inside the exposure chamber was analyzed frequently and revealed a CS concentration of 800–1000 mg/m^3^ total suspended particles and a carbon monoxide concentration of 300–800 ppm. Age-matched WT and RAGE^-/-^ mice exposed to room-air were used as controls.

### Long-term cigarette smoke exposure

To analyze a CS induced lung damage, eight week old female WT and RAGE^-/-^ mice were exposed to the smoke of 3R4F cigarettes (College of Agriculture, Reference Cigarette Program, University of Kentucky, Lexington, Kentucky, USA) in a TE-10 smoking machine (Teague Enterprises, Woodland, California, USA) [27]. Mice were subjected to CS for 261 minutes /day, five days/week for six months. The daily smoking protocol consists of three smoking phases each with 87 minutes, which were interrupted by room-air exposures for 40 min. The concentration of CS was 120 mg/m^3^ total suspended particles. Age-matched WT and RAGE^-/-^ mice exposed to room-air were used as controls.

### Pulmonary function measurements, stereological analysis, and immunohistochemistry

For the evaluation of respiratory system mechanics, mice were analyzed using the FlexiVent system (Scireq, Montreal, Canada) for forced oscillation measurements as described earlier [27, 28]. Mice were anesthetized with ketamine (7 mg/kg, i.p.) and xylazine hydrochloride (105 mg/kg, i.p.). Once the anesthesia was reached, the trachea was cannulated and the animals were attached to the FlexiVent apparatus. For data acquisition we used the flexiWare 7.1 (Scireq Inc., Montreal, Canada) software.

After invasive pulmonary function measurements, mice were euthanized and the lungs were excised and prepared for systematic uniform random sampling for stereological analysis. Lungs were fixed by instillation of phosphate buffered saline (PBS) -buffered 4% formalin under a constant hydrostatic pressure of 30 cm for 15 minutes and placed in PBS-buffered 4% formalin for 24 hours. The fixed lungs were embedded in 1% agarose and cut into regular slices of exactly the same thickness and embedded in paraffin. The mean chord length was calculated using the Visiopharm Integrator System (Visiopharm, Hoersholm, Denmark) on an Olympus BX51 microscope equipped with an 8-position slide holder. Hematoxilin and eosin staining was done following standard protocols [27].

For immunohistochemistry formalin-fixed and paraffin-embedded lung slides were incubated with a polyclonal goat-anti RAGE antibody (AF-1179, R&D systems, Wiesbaden-Nordenstadt, Germany) and visualized by an AEC-staining kit (Sigma-Aldrich, Steinheim, Germany).

### Bronchoalveolar lavage fluid collection and differential cell count

Collection of BALF was performed as previously described [29]. Briefly, mice were euthanized, the tracheae were cannulated and the lungs were rinsed three times with 1 mL PBS. BALF were centrifuged at 300 g for 10 minutes to obtain BAL cells and cell-free supernatants. The cell pellets were resuspended in an appropriate volume of PBS, total cell numbers were determined and differential cell counts were done on cytospin preparations after staining with Diff-Quick (Medion Diagnostic, Gräfelfing, Germany).

### Isolation and ex vivo stimulation of alveolar macrophages

Murine alveolar macrophages were isolated from ten to eleven weeks old RAGE^-/-^ and WT mice by flushing the lungs ten times with 1 mL PBS supplemented with 0.5 mM EDTA, respectively. For the cultivation, all cells of each group were pooled and centrifuged at 800 rpm for 5 minutes. The alveolar macrophages were recovered in RPMI 1640 (Invitrogen, Grand Island, NY, USA) supplemented with 10% fetal calf serum (FCS, Invitrogen, Grand Island, NY, USA), 100 U/mL penicillin, and 100 U/mL streptomycin (PAA Laboratories GmbH, Pasching, Austria) and seeded at a density of 1x10^5^ / well in 48-well plates. After two hours, non-adherent cells were removed by changing the medium.

To analyze the inflammatory capacity, alveolar macrophages were classically activated as previously described [30]. In brief, 24 hours after isolation the cells were primed with 40 ng/mL IFNɣ (biolegend, Fell,Germany) for 16 hours. In the next step, IFNɣ was removed and the cells were stimulated with 10 ng/mL lipopolysaccharide (LPS) or TLR2/6 ligand Pam2CSK4 (invivogen, Toulouse, France) for 24 hours.

### Isolation and ex vivo cultivation of murine alveolar epithelial cells

Alveolar epithelial cells were isolated from lungs of ten week old female WT and RAGE^-/-^ mice following a protocol adapted to the isolation procedure of primary human alveolar epithelial cells [31]. In brief, mice were euthanized, thoraces were opened and the lungs were perfused with PBS until they were visually free of blood. A 22-gauge catheter was inserted into the trachea and 2 mL dispase solution (Corning, New York, USA) was administered to the airways. After excision of the lung, the tissue was incubated in another 2 mL of dispase at 37°C for 45 minutes. The partially digested lungs were transferred into a culture dish containing 10 mL CMM (complete mouse medium: DMEM/F12 supplemented with 1mM L-glutamine, 10 mM HEPES (life technologie, Carlsbad, California, USA), 0.25% bovine serum albumin, 1% non-essential amino acids (Sigma-Aldrich, Steinheim, Germany), 100 μg/mL primocin (invivogen, Toulouse, France), 0.05% insulin-transferrin-sodium selenite (Roche, Basel, Switzerland)) plus 1 mL DNase (Sigma-Aldrich, Steinheim, Germany) [32]. Tissue was chopped into small pieces and incubated at 37°C for 45 minutes. The received cell suspension was filtered, centrifuged and the cell pellet was resuspended in 20 mL CMM plus 1 mL DNase and transferred to culture dishes. In a 90-minutes incubation step at 37°C, macrophages were removed by adhesion to the plastic surface whereas the epithelial cells remained in solution. The supernatants were collected, centrifuged and the cell pellet was resuspended in BSSB buffer. Afterwards the cells were incubated with an anti-CD326 PE antibody (ebioscience, San Diego, California, USA) at 4°C for 30 minutes followed by a second incubation step with anti-PE MicroBeads (Miltenyi Biotec, Bergisch Gladbach, Germany). Finally, the cell suspension was transferred to a magnetic MACS column (Miltenyi Biotec, Bergisch Gladbach, Germany) and the CD326-positive cells were eluted with 5 mL CMM plus 10% FBS (PAA Laboratories GmbH, Pasching, Austria). 5x10^5^ cells were seeded on the upper side of a laminin- and fibronectin-coated transwell filter insert (0.33 cm^2^, pore size 0.4 μm, Corning, New York, USA). After 48 hours, cells reached 100% confluence and the apical medium was removed to promote the polarization of the cells.

When monolayers reached their highest transepithelial electrical resistance (TEER) 48 hours after airlift, alveolar epithelial cells were used for CS experiments following an *in vitro* exposure protocol as previously described [33]. Briefly, smoke generated from three unfiltered 3R4F cigarettes (College of Agriculture, Reference Cigarette Program, University of Kentucky, Lexington, Kentucky, USA) was guided into an airtight exposure chamber containing the tissue cultures. Directly after the exposure, medium in the basolateral compartment was renewed and the cells were cultured under humidified atmosphere of 5% CO_2_ and 37°C for 24 hours.

### Cytokine, albumin, and nitrite measurements

KC, interleukin (IL)-6 and tumor necrosis factor α (TNFα) in BALFs or cell supernatants were measured using an ELISA development kit by R&D systems (Wiesbaden-Nordenstadt, Germany) on a Fluostar Omega ELISA reader (BMG Labtech, Ortenberg, Germany). Albumin in BALF was analyzed with a mouse albumin ELISA Quantitation Kit (Bethyl Laboratories, Montgomery, Texas, USA). A Griess Reagent Kit from Molecular Probes (Eugene, Oregon, USA) was used for determination of nitrite (NO_2_^-^) in cell supernatants of alveolar macrophages.

### Quantitative RT-PCR (qRT-PCR)

Total RNA from lung tissue was isolated using the Trizol Reagent (Life Technologies, Carlsbad, California, USA) and RNA from alveolar epithelial cells was obtained with a NucleoSpin^®^ RNA Set (Macherey-Nagel, Düren, Germany). A cDNA synthesis Kit from Thermo Scientific (Waltham, Massachusetts, USA) was used for reverse transcription. qRT-PCRs were performed with a SYBR Green Kit (Bioline, Luckenwalde, Germany). Primers were as follows: 18S: 5- GTA ACC CGT TGA ACC CCA TT-3’ and 5’-CCA TCC AAT CGG TAG TAG CG-3’; Occludin: 5’-CCC TGA AAT ACA AAG GCA-3’ and 5’-GAG TTA ACG TCG TGG ACC-3’; zonula occludens (Zo)-1: 5’-GGC ATT CCT GCT GGT TAC-3’ and 5’-AGG ACA CCA AAG CAT GTG-3’ [34]; Claudin 18 (Cldn18): 5‘- GAC CGT TCA GAC CAG GTA CA-3‘ and 5‘-GCG ATG CAC ATC ATC ACT C-3‘; aquaporin-5 (AQP5): 5’ CTG CTC CGA GCC ATC TTC TA-3’ and 5’-GGT GAA GTA GAT CCC CAC AAG A-3’; surfactant protein–C (SP-C): 5’-CAC AGC AAG GCC TAG GAA AG-3’ and 5’-ATC CAA CCC AGT CCC TCT CT-3’.

### SDS-PAGE and western blot

Primary alveolar epithelial cells were isolated and cultivated as described above. SDS-PAGE and Western blot were performed as described earlier [35]. The following antibodies were used: Claudin-18 (38–8000, ThermoFisher, 1:500), SP-C (ab90716, Abcam, 1:500), AQ5 (ab92320, Abcam, 1:500), and a-Tubulin (ab89984, Abcam, 1:1000), anti-rabbit-HRP (P0448, DakoCytomation, Glostrup, Denmark, 1:1000), and anti-chicken-HRP (ab97135, Abcam, 1:1000). HRP-activity was visualized with Clarity Western ECL substrate (BioRad, Hercules, USA). The membranes were developed on conventional films in a dark room and analyzed densitometrically after scanning with TINA 2.0 (Raytest GmbH, Germany).

### Statistical analysis

Comparisons between two groups were analyzed by the Student’s t-test (two-sided) or Mann-Whitney test when data were not normally distributed. For normality test, we used the D’Agostino and Pearson omnibus test followed by a one-sample test. Two-way ANOVA with Bonferroni adjustment was used for TEER kinetics in alveolar epithelial monolayers. Results were considered statistically significant for p < 0.05. All statistical tests were performed using the software Prism (GraphPad Software, San Diego, California, USA).

## Results

### RAGE is expressed on alveolar epithelial cells and alveolar macrophages

**Fig 1:**
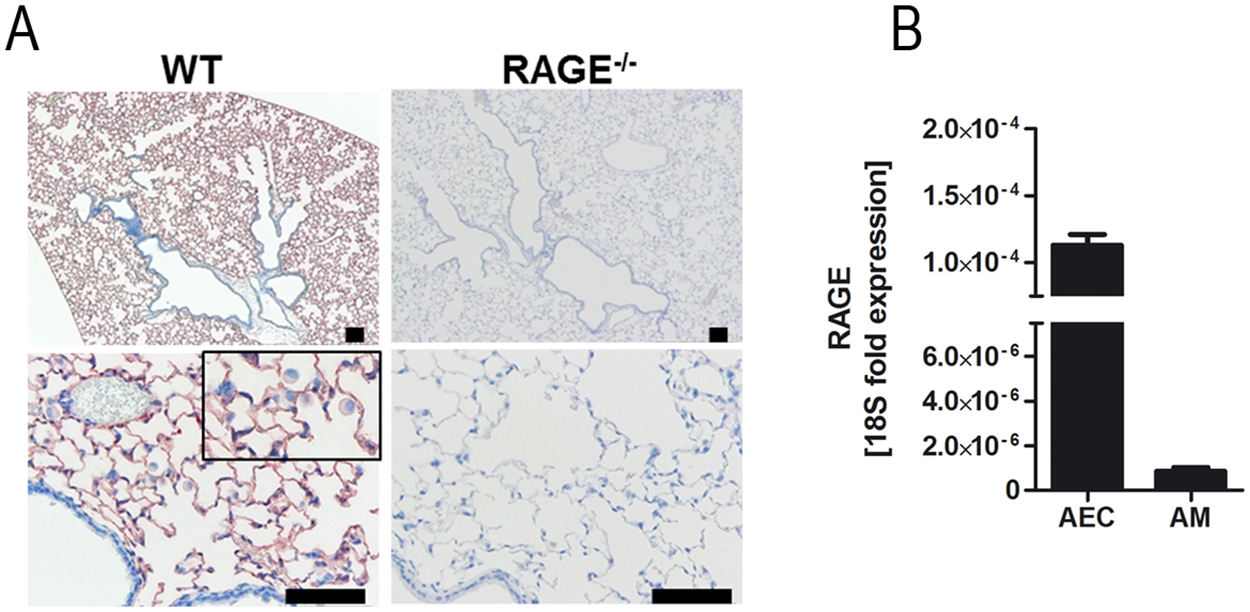
RAGE is expressed on alveolar epithelial cells and alveolar macrophages. (A) Immunostaining for RAGE protein in WT and RAGE^-/-^ lung tissue. (B) Isolated alveolar epithelial cells (AEC) (n = 7 per group) and alveolar macrophages (AM) (n = 4 per group) were analyzed for RAGE mRNA expression using qRT-PCR. Data are shown as mean ± SEM. Scale bar: 100μm.

To characterize the expression pattern of RAGE, sections of lung tissue were subjected to immunohistochemistry. In lungs of WT mice, immunohistochemical staining with anti-RAGE-antibody revealed a strong signal in the alveolar epithelium and alveolar macrophages, whereas no staining could be detected in bronchi, bronchioles, endothelial cells and connective tissue (Fig 1A). qRT-PCR on isolated murine alveolar epithelial cells and alveolar macrophages confirmed the RAGE expression in these cells and indicated higher mRNA levels in alveolar epithelial cells as in alveolar macrophages (Fig 1B).

### RAGE has a role in the maintenance of pulmonary mechanics and structure

**Fig 2:**
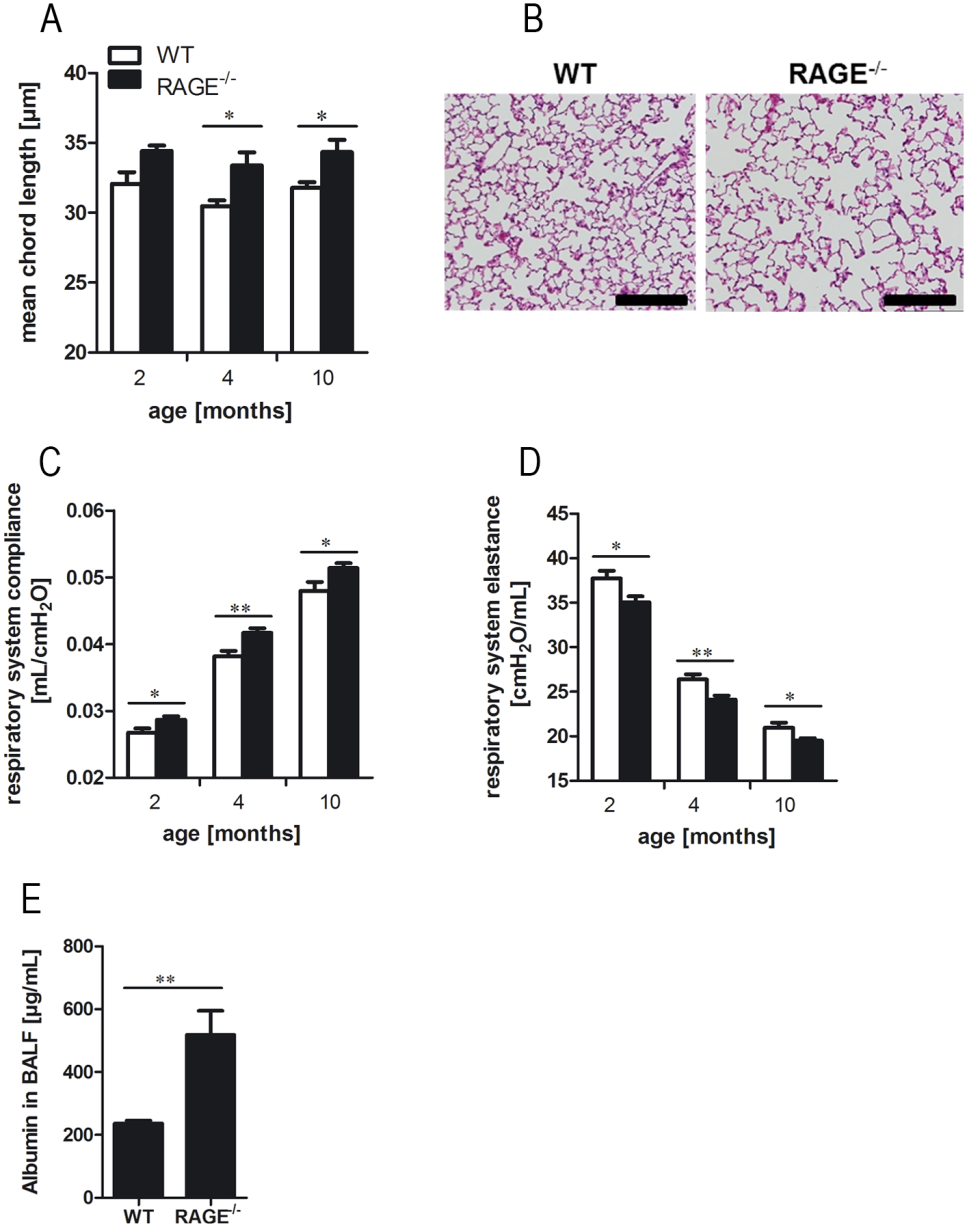
RAGE contributes to maintenance of pulmonary mechanics and structure. Quantification of mean chord length (A) were performed by stereological analysis of alveolar parenchyma; n ≥ 4 per group. (B) Representative histology (hematoxylin and eosin staining) of ten months old WT and RAGE^-/-^ mice; scale bars: 200μm. Respiratory system compliance (C) and respiratory system elastance (D) were determined in two, four and ten months old WT and RAGE^-/-^ mice using invasive pulmonary function measurements; n ≥ 7 per group. (E) Concentration of serum protein albumin in BALF of two months old mice; n = 10. Data are shown as mean ± SEM. *p < 0.05 and **p < 0.01.

To evaluate the impact of RAGE on the maintenance of pulmonary mechanics and structure, WT and RAGE^-/-^ mice of different ages were studied in functional and stereological analysis. In four and ten month old mice, RAGE deficiency caused an altered pulmonary structure as shown by the mean chord length (Fig 2A) and alveolar histology (Fig 2B). Measurements of invasive pulmonary function revealed an effect of RAGE deficiency on the pulmonary mechanics. The respiratory system compliance (Fig 2C) was significantly increased in RAGE^-/-^ mice, whereas the respiratory system elastance (Fig 2D) was significantly decreased as compared to WT mice. With advanced age of WT and RAGE^-/-^ mice the respiratory system compliance increased and the respiratory system elastance decreased, but the differences between the mouse strains were age-independent. To analyze whether RAGE modulates the alveolar-blood barrier, we determined the concentrations of serum protein albumin in BALF and found significantly increased albumin levels in RAGE^-/-^ mice as compared to WT animals (Fig 2E).

### RAGE regulates differentiation and barrier formation of isolated alveolar epithelial cells

**Fig 3:**
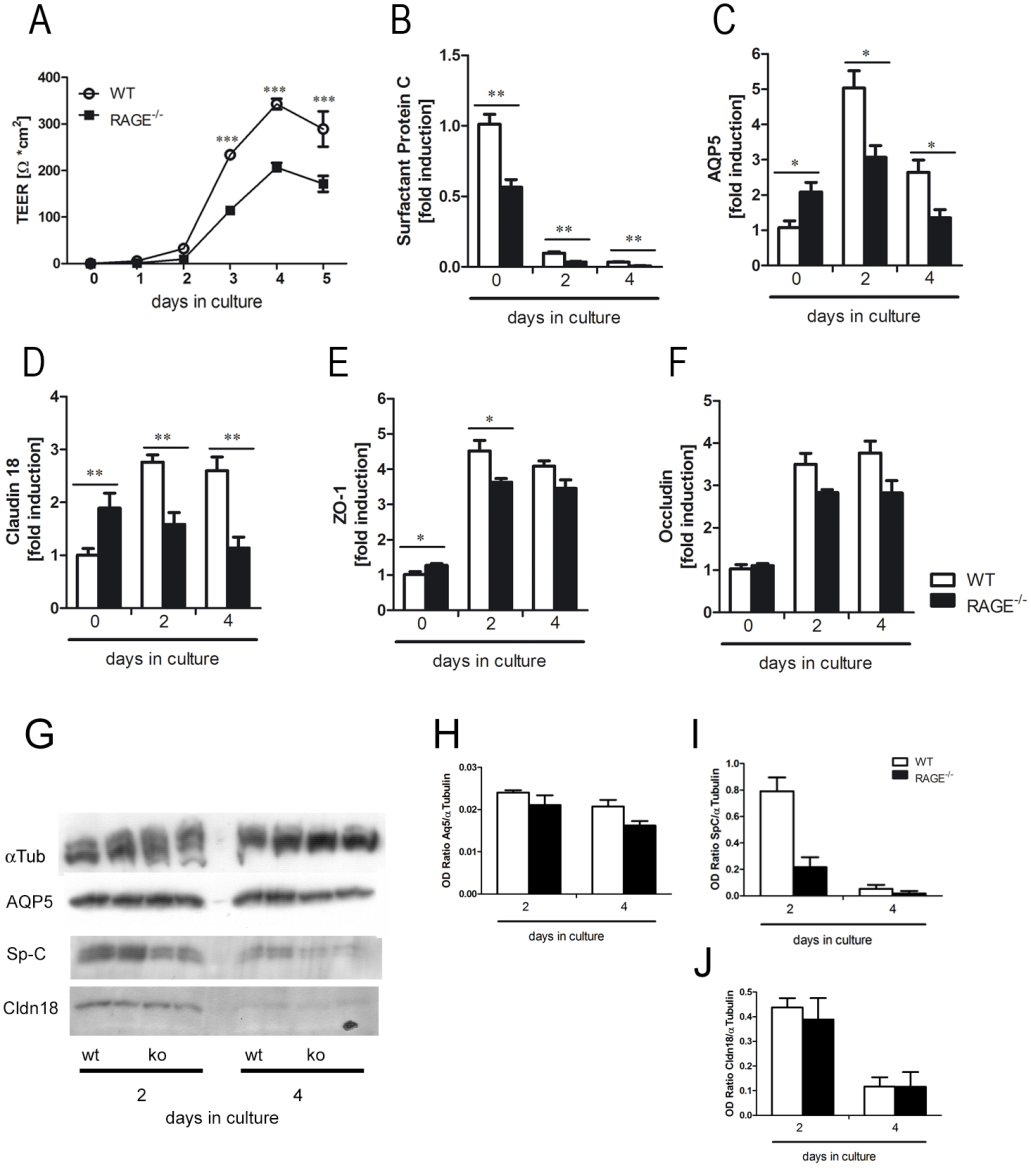
RAGE promotes the differentiation and formation of an intact barrier function in primary murine alveolar epithelial cells. Alveolar epithelial cells were isolated from WT and RAGE^-/-^ mice and analyzed in an air-liquid interface culture system. (A) TEER of isolated alveolar epithelial cells was measured for a period of five days; n = 12 per group. Relative mRNA induction of alveolar epithelial type 2 cell marker surfactant protein C (B), alveolar epithelial type 1 cell marker aquaporin-5 (AQP5) (C) and the tight junction proteins claudin 18 (D), ZO-1 (E) and occludin (F) was determined by qRT-PCR; n = 6 per group. The proteins of cell lysate of alveolar epithelial cells at day 2 and 4 post isolation were separated by SDS-PAGE and stained with antibodies against α-Tubulin (αTub), aquaporin-5 (AQP5), pro-surfactant protein C (Sp-C), and claudin 18 (Cldn18) (G). The bands of the blot were densitometrically analyzed and the results are shown for AQP5 (H), SP-C (I), and Cldn18 (J). Data are shown as mean ± SEM. *p < 0.05; **p < 0.01 and ***p < 0.001.

As RAGE deficiency resulted in loss of lung structure *in vivo*, we characterized the role of RAGE in differentiation and barrier formation of primary murine alveolar epithelial cells *ex vivo*. Measurement of TEER during the differentiation of primary alveolar epithelial cells revealed significantly decreased TEER values in alveolar epithelial cells isolated from RAGE^-/-^ mice (Fig 3A). qRT-PCR on different differentiation markers and tight junctions proteins revealed a continuously decreased expression of the alveolar type 2 cell marker surfactant protein C (SP-C) during cultivation in both groups (Fig 3B). WT cells showed an increased expression of alveolar type 1 cell marker AQP5 with a peak at the second day in culture. There was no significantly increased expression of AQP5 in RAGE^-/-^ cells (Fig 3C). The expression of claudin 18 was significantly increased in freshly isolated RAGE^-/-^ cells as compared to WT cells. During the differentiation phase the expression of claudin 18 (Cldn18) was significantly decreased in RAGE^-/-^ cells, whereas a significant increase could be detected in WT cells (Fig 3D). ZO-1 (Fig 3E) and occludin (Fig 3F) expression increased during the differentiation phase in both groups. However, RAGE deficiency resulted in a significantly lower ZO-1 expression at the second day in culture (Fi. 3E) and a decreased expression of occludin at the fourth day in culture as compared to the WT cells. These data show that RAGE is involved in the regulation of the epithelial barrier function. To support the gene expression data we analyzed cell lysate from isolated alveolar epithelial cells at day 2 and 4 post isolation for some of the markers shown above (Fig 3G). The densitometric quantification of the western blots showed equivalent results for AQP5, SP-C, and Cldn18 as the qRT-PCR (Fig 3H–3J).

### RAGE modulates the inflammatory response in alveolar macrophages

**Fig 4:**
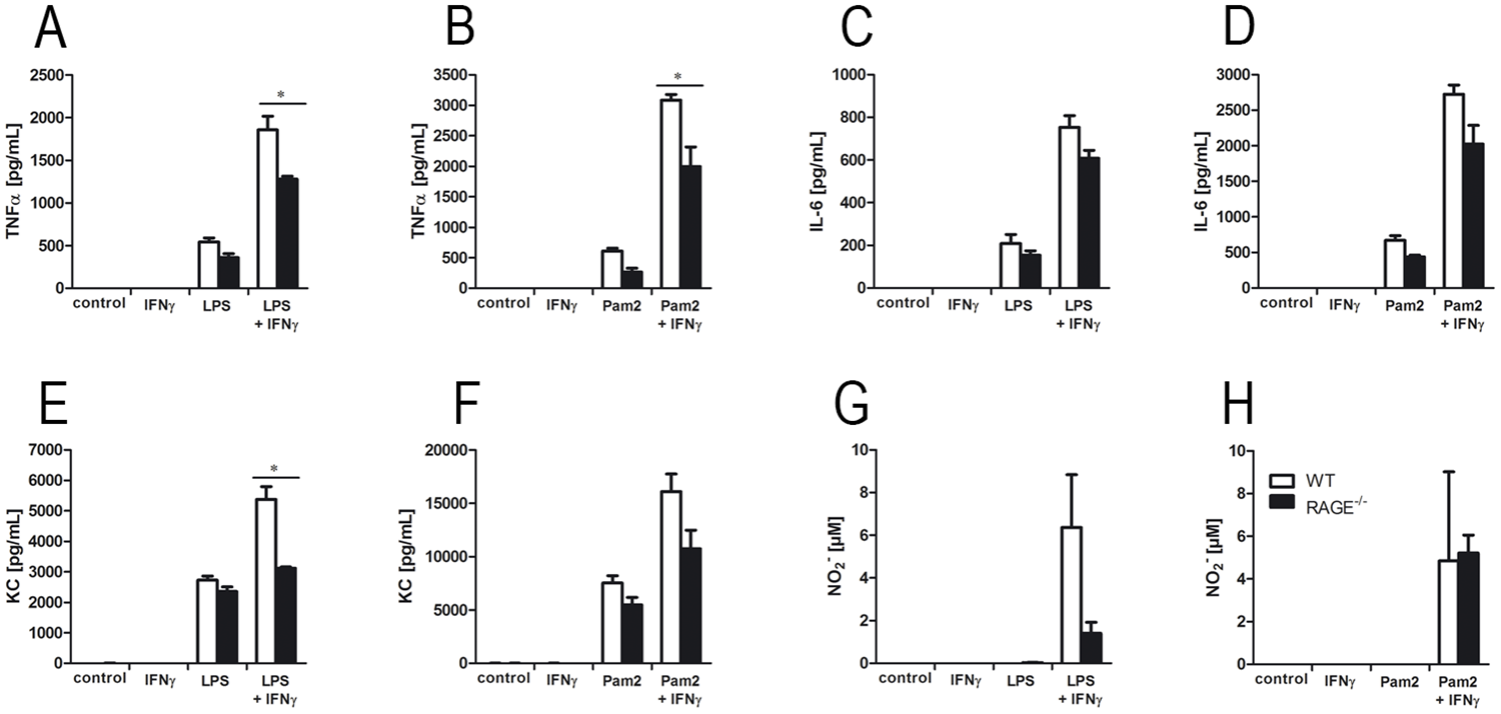
RAGE modulates the inflammatory response in alveolar macrophages. Alveolar macrophages were isolated from lungs of WT and RAGE^-/-^ mice. After activation with IFNɣ for 16 hours, alveolar macrophages were stimulated with TLR ligands LPS or Pam2CSK4 (Pam2) for 24 hours. Concentrations of TNFα (A and B), IL-6 (C and D), KC (E and F) and NO_2_^-^ (G and H) were determined in supernatants; n ≥ 3 per group, representative for three independent experiments. Data are shown as mean ± SEM. *p < 0.05; **p < 0.01 and ***p < 0.001.

To examine the impact of RAGE deficiency on inflammatory responses of macrophages, we isolated alveolar macrophages from WT and RAGE^-/-^ mice and stimulated the cells with IFNγ, the TLR ligands Pam2CSK4 and/or LPS [30]. Concentrations of TNFα (Fig 4A and 4B), IL-6 (Fig 4C and 4D) and KC (Fig 4E and 4F) increased after LPS and Pam2CSK4 stimulation. A previous activation with IFNγ resulted in an additional cytokine release in response to Pam2CSK4 or LPS. However, in alveolar macrophages obtained from RAGE^-/-^ mice TNFα and KC release was significantly decreased after IFNγ and TLR stimulation as compared to WT cells. Detection of NO_2_^-^ by using Griess reagent showed an increase of NO_2_^-^ levels after IFNɣ and TLR ligand application, but revealed no significant differences between the groups (Fig 4G and 4H).

### RAGE promotes the development of CS-induced lung damage

**Fig 5:**
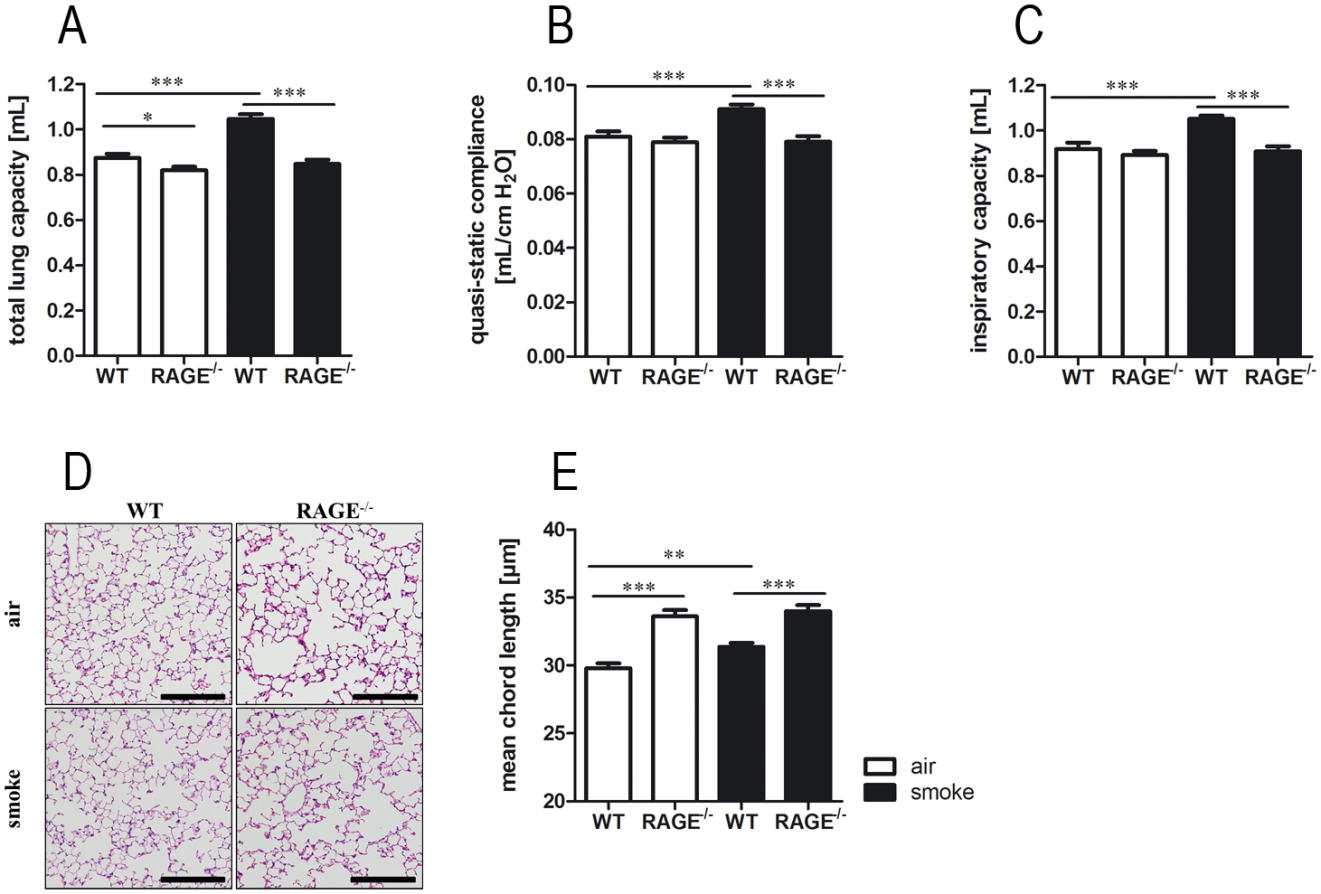
RAGE promotes the development of CS-induced lung damage. WT and RAGE^-/-^ mice were exposed to CS or room-air for six months. Total lung capacity (A), quasi-static compliance (B) and inspiratory capacity (C) were determined by invasive pulmonary function measurements; n ≥ 18 per group. (D) Representative histology of alveolar parenchyma (hematoxylin and eosin staining); scale bars: 100μm. Quantification of mean chord length (E) were performed by stereological analysis of alveolar parenchyma; n ≥ 10 per group. Data are shown as mean ± SEM. *p < 0.05; **p < 0.01 and ***p < 0.001.

To analyze the role of RAGE in response to chronic CS exposure, WT and RAGE^-/-^ mice were exposed to CS or room air for six months. Invasive pulmonary function measurements revealed characteristic smoke-induced changes of respiratory system mechanics only in WT mice. The total lung capacity (Fig 5A), quasi-static compliance (Fig 5B), and inspiratory capacity (Fig 5C) were significantly increased after chronic CS exposure in WT mice, whereas CS-exposed RAGE^-/-^ mice showed no differences as compared to control animals. Histopathologic (Fig 5D) and stereologic analysis of alveolar parenchyma revealed a CS-induced increase of mean chord length in WT mice (Fig 5E). Room-air exposed RAGE^-/-^ mice showed a significantly increased mean chord length as compared to WT mice (Fig 5D). In these mice CS exposure had no further impact on airspace enlargement.

### RAGE regulates inflammatory response to acute CS exposure

**Fig 6:**
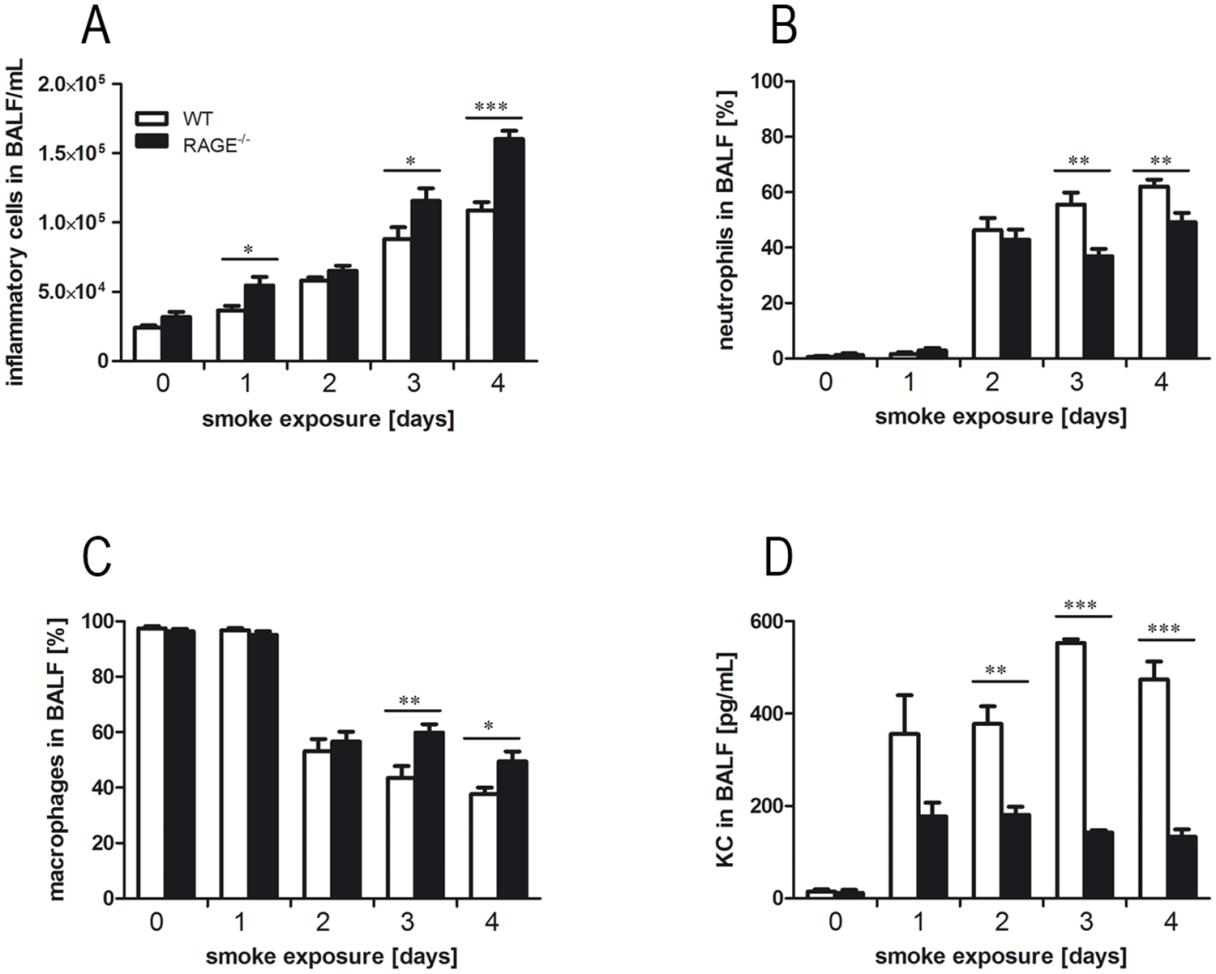
RAGE modulates inflammatory processes resulting from acute CS exposure. WT and RAGE^-/-^ mice were exposed to acute CS exposure for four days. Numbers of total inflammatory cells (A), neutrophils (B) and macrophages (C) were determined in BALF of WT and RAGE^-/-^ mice; n ≥ 9 per group. Concentrations of KC in BALF were determined using ELISA (D); n ≥ 9. Data are shown as mean ± SEM. *p < 0.05; ***p < 0.001.

To study whether RAGE is involved in the inflammatory response to acute CS exposure, inflammatory cells and the chemokine KC were analyzed in lungs of WT and RAGE^-/-^ mice. In both groups, the numbers of inflammatory cells increased during CS exposure. After one, three and four days of CS exposure, RAGE^-/-^ mice showed significantly higher cell numbers as compared to WT animals (Fig 6A). Furthermore, differential cell counts revealed different cellular compositions in BALF of RAGE^-/-^ and WT mice. Acute CS exposure resulted in a percentage increase of BALF neutrophils in both groups (Fig 6B). However, RAGE^-/-^ mice showed significantly lower percentages of neutrophils after three and four days of CS exposure as WT mice. At these time points RAGE^-/-^ mice exhibited significantly increased percentage of BALF macrophages compared to WT mice (Fig 6C). Pulmonary concentrations of KC were significantly reduced in RAGE^-/-^ mice after two, three and four days of CS exposure as compared to WT mice (Fig 6D).

## Discussion

The main finding of the present study is that RAGE participates in the maintenance of pulmonary mechanics and structure. We show that RAGE regulates the differentiation of alveolar epithelial cells and is involved in regulation of the alveolar barrier function. In CS-induced lung pathology, RAGE-mediates inflammation and has a role in subsequent damage of the lung structure.

In this study we used two smoke exposure models that address different aspects of COPD. In the acute model the inflammation is mainly based on the recruitment of neutrophilic granulocytes from the circulation while the chronic model predominantly causes a macrophage based inflammation and additional tissue damage. Both cell types have distinct functions and consequences in lung physiology and secrete different mediators that may interact with RAGE. Based on the technical features of the smoking exposure machines, two different setups were necessary to apply both conditions.

Under physiological conditions, RAGE is highly expressed in the lung, whereas it shows only low amounts of mRNA expression in other organs [2]. The analysis of pulmonary function and lung structure allows obtaining the physiological function of RAGE in the pulmonary system. Invasive lung function measurements and stereological analysis of lung parenchyma revealed that RAGE participates in maintenance of lung structure. RAGE^-/-^ mice showed increased respiratory system compliance and a decreased respiratory system elastance as compared to WT mice. Moreover, RAGE deficiency spontaneously resulted in a loss of lung structure in native mice of different age. Our data are in line with other *in vivo* studies using RAGE^-/-^ mice. Al-Robaiy et al. assessed pulmonary biomechanics by *ex vivo* lung function measurements. In their study, lungs of RAGE^-/-^ mice showed a decreased expiratory airflow and increased lung compliance as compared to WT mice. However, they could only define an impact of RAGE on the respiratory function in mice from an age of five months, whereas we could detect an influence even in younger mice [36]. Sambamurthy et al. observed comparable enlargement of alveolar dimensions in native RAGE^-/-^ mice [37]. The RAGE-dependent differences in lung function and structure could be caused by insufficient cell-cell- and cell-matrix contacts. RAGE mediates interactions between two adjacent cells and enhances the adherence of alveolar epithelial cells to the basal membrane [2, 36].

In addition to the changes in pulmonary function and structure, RAGE deficiency also affects the alveolar epithelial barrier. Naive RAGE^-/-^ mice had significantly increased concentrations of albumin in their BALF. Leakage of serum proteins from the blood into the lung indicates an endothelial-epithelial barrier defect in RAGE^-/-^ mice [34, 38–40]. Other studies using increased total protein content in the BALF of mice as a marker for barrier dysfunction could not determine any differences between WT and RAGE^-/-^ mice [36, 41, 42].

Alveolar epithelial cells are the main source of pulmonary RAGE expression [2, 3] and have a critical role in maintaining the pulmonary barrier function [43, 44]. Therefore we analyzed the barrier function with TEER of primary murine alveolar epithelial cells under *ex vivo* conditions. TEER measurement of alveolar epithelial monolayers revealed significantly decreased values in RAGE^-/-^ cells. This is in line with the observed barrier defect in the *in vivo* experiment. In addition, RAGE deficiency affected the differentiation of alveolar epithelial cells *ex vivo*. Primary alveolar epithelial cells cultivated on transwell inserts acquire characteristics of the alveolar type 1 cells [2, 32, 45, 46]. Therefore, the expression of alveolar type 2 cell markers like surfactant protein C or D is downregulated whereas mRNA levels of alveolar type 1 marker AQP5 or caveolin-1 increases [2, 32]. In our study, the expression of surfactant protein C decreased during the differentiation of the cells. However, AQP5 showed a significantly increased expression only in RAGE-producing cells, which indicates a potential role of RAGE in differentiation of alveolar epithelial cells. In addition, RAGE^-/-^ cells showed an altered expression profile of claudin 18. The analysis of the protein levels of the above mentioned markers correlated with the gene expression. The tight junction protein claudin 18 is only expressed on alveolar epithelial cells [47]. Two independent studies showed that claudin 18 is of crucial importance to development and maintenance of alveolar epithelial barrier [48, 49]. Mice deficient for claudin 18 revealed an increased alveolar permeability for fluorescence-labelled tracer molecules and showed incomplete cell-cell-contacts between adjacent alveolar type 1 cells [48, 49]. Furthermore, measurement of the TEER of primary alveolar epithelial cells deficient for claudin 18 showed decreased values comparable to our *in vitro* results of RAGE deficient cells [49]. The function of tight junction complexes can be regulated in multiple ways and is not strictly bound to de novo protein synthesis [50, 51].

Since alveolar macrophages are the second cellular source of pulmonary RAGE expression, we further characterized the impact of RAGE deficiency on this cell type. *Ex vivo* stimulation with IFNɣ in combination with LPS or Pam2CSK4 resulted in increased secretion of TNFα, KC, IL-6 and NO_2_^-^ that was significantly reduced in RAGE^-/-^ macrophages. These findings are in line with studies that show that RAGE signaling induces inflammation by activating downstream ligands and signaling cascades [52–55] that predominantly lead to the activation of NF-κB dependent pro-inflammatory responses. Another study with RAGE^-/-^ alveolar macrophages stimulated with CS extract showed significantly decreased levels of TNFα and IL-1β [24]. Furthermore, Chen et al. demonstrated a participation of RAGE in CS induced nitric oxide generation *in vitro* [56]. It has also been shown that the blocking of RAGE results in reduced TNFα secretion and lower phosphorylation of NF-κB [53]. All these data underscore that RAGE in alveolar macrophages is necessary for induction and modulation of an inflammatory response.

As RAGE shows high expression levels in lung tissue of smokers and COPD patients [20, 56, 57] and is correlated with disease severity [58, 59], we further analyzed the role of RAGE in CS exposure models. Chronic CS exposure of mice leads to lung function decrease and structural lung damage [27, 60, 61]. In our chronic CS model, emphysema-like changes were not further increased in RAGE^-/-^ mice. This has also been found by Waseda et al., who analyzed RAGE-dependent lung damage in a murine COPD elastase model. Intratracheal instillation of elastase resulted in a significantly increase of static lung compliance in WT mice, whereas no effect could be observed in RAGE^-/-^ mice [22]. In another CS-exposure study, structural changes of lung parenchyma were only detected in WT mice. RAGE^-/-^ mice revealed significantly increased alveolar dimension at baseline, but showed no additional changes after CS exposure. In the same study, pulmonary function measurement after methacholine challenge showed no differences in the pulmonary function between WT and RAGE^-/-^ mice [37]. In our acute CS exposure model, RAGE deficiency was associated with an altered response to CS inhalation. RAGE^-/-^ mice showed significantly higher leukocyte numbers, which were mainly attributable to an influx of macrophages. Furthermore, as in the *ex vivo* stimulation experiment with primary alveolar macrophages, concentrations of the chemokine KC were significantly reduced in RAGE^-/-^ mice.

In summary, the present data show that RAGE is important for pulmonary mechanics and structure and is involved in the development of CS-induced pathology. RAGE participates in differentiation of alveolar epithelial cells and is required for the development of an intact pulmonary structure and alveolar barrier integrity. In alveolar macrophages, RAGE is involved in the regulation of inflammatory processes that mediates CS-induced alveolar tissue destruction. Together with the available data from clinical investigation, RAGE has a complex role in lung physiology and the response of the lung to noxious exposures.
